# Does brain activity cause consciousness? A thought experiment

**DOI:** 10.1371/journal.pbio.3001651

**Published:** 2022-06-10

**Authors:** Albert Gidon, Jaan Aru, Matthew Evan Larkum

**Affiliations:** 1 Institute of Biology, Humboldt University Berlin, Berlin, Germany; 2 Institute of Computer Science, University of Tartu, Tartu, Estonia; 3 Neurocure Center for Excellence, Charité Universitätsmedizin Berlin, Berlin, Germany; University of Amsterdam: Universiteit van Amsterdam, NETHERLANDS

## Abstract

Rapid advances in neuroscience have provided remarkable breakthroughs in understanding the brain on many fronts. Although promising, the role of these advancements in solving the problem of consciousness is still unclear. Based on technologies conceivably within the grasp of modern neuroscience, we discuss a thought experiment in which neural activity, in the form of action potentials, is initially recorded from all the neurons in a participant’s brain during a conscious experience and then played back into the same neurons. We consider whether this artificial replay can reconstitute a conscious experience. The possible outcomes of this experiment unravel hidden costs and pitfalls in understanding consciousness from the neurosciences’ perspective and challenge the conventional wisdom that causally links action potentials and consciousness.

## Abstract 2

The authors of this Essay examine whether action potentials cause consciousness in a three-step thought experiment that assumes technology is advanced enough to fully manipulate our brains.

## Introduction

The idea of stimulating the brain to evoke conscious experiences has a long history in neuroscience [1–4]. Nowadays, brain–machine interfaces [5] encode and decode neuronal activity [6–8] and are routinely used to control neuroprosthetics [9]. Electrical stimulation of sensory brain areas is becoming sufficiently precise to deliver specific content, bypassing sensory organs [10] or diseased brain tissue [11]. Furthermore, it is now possible to evoke a memory by selectively reactivating ensembles of neurons (i.e., the engram) that were naturally active in the animal’s brain in a previous event [12,13] (for review, see [14]). Although brain activity can take many forms, it is almost always associated with the neuronal firing of action potentials. Moreover, the effective use of action potentials in brain–machine interfaces with neuroprosthetics and rehabilitation of neural function [5] suggests that action potentials are the fundamental unit of information in the brain.

In experiments routinely performed in neurobiological laboratories, action potentials are recorded and evoked in single neurons and even in small-scale networks [15,16] using current clamp and voltage clamp techniques. Using these techniques, triggering action potentials at the researcher’s bidding (rather than naturally due to the synaptic inputs) is commonplace and even mundane in a modern electrophysiological laboratory. The rapid development of tools and technologies in neuroscience [17–21] brings the goal of capturing every action potential in every neuron of the brain ever closer [22,23]. To date, the highest number of channels recorded by an electrode array belongs to the Argo system, with 65,536 channels [24]. These technologies provide unprecedented insights into the fine details of brain function. Thus, it is perhaps just a matter of time until newer, more powerful technologies will eventually allow us to solve the mechanics of how the brain works. As we converge on this goal, will we get closer to understanding brain function and, with it, the biological causes of conscious experience?

The fact that there is no commonly accepted definition of consciousness has not prevented researchers from pursuing the neural mechanisms underlying consciousness [25,26]. Here, we took the approach that it is sufficient to identify the target of the investigation rather than strictly define it [27]: Consciousness is the experience of ourselves and the surroundings that fades when we enter deep sleep or under anesthesia (cf [28,29]). In a typical experimental paradigm to study consciousness, a visual stimulus is briefly presented to a participant. The stimulus is constructed to be consciously perceived in some trials and not in others [30–32]. Contrasting neural activity of these 2 types of trials allows researchers to delineate the neural processes underlying consciousness (with some caveats, see [30,31]). The research of the fundamental questions regarding the mechanisms and functions of consciousness has also benefited us with new tools to diagnose disorders related to consciousness [33–35].

**Fig 1:**
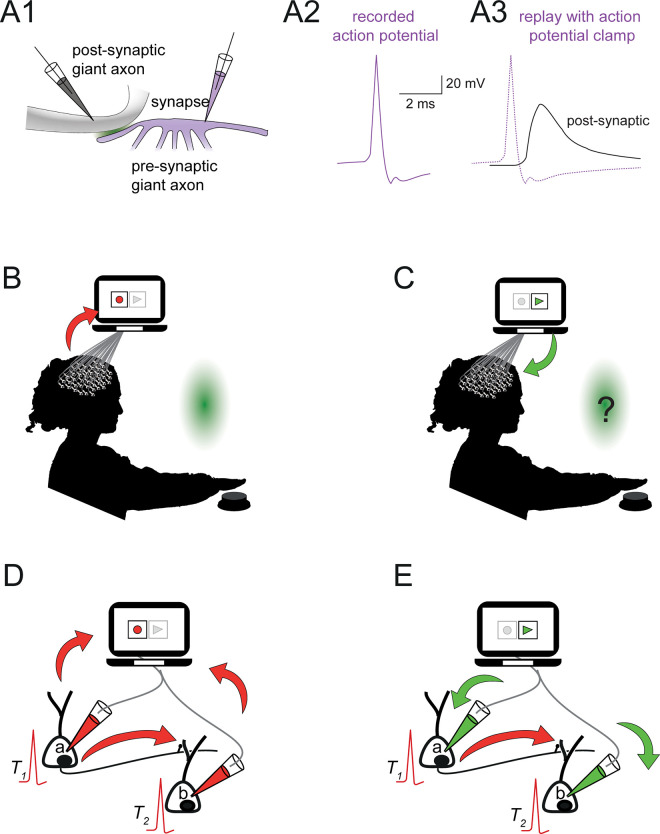
Recording and replaying action potentials in the entire brain. **(A1)** Experimental schematic of the action potential replay in the squid giant synapse as done in [41]. **(A2)** Action potential recorded from the presynaptic terminal (solid purple line) is set as the “command” voltage. **(A3)** The action potential recorded in A2 (i.e., the command voltage) is replayed (dotted purple line), and the postsynaptic response was “virtually indistinguishable from that obtained with the original presynaptic spike activation” [42]. **(B)** A stimulus (green light on a screen) is presented to the participant while all action potentials from each neuron in the brain are recorded and stored for later retrieval. The participant is asked to press a button when she consciously perceives the green light. **(C) Step 1:** All patterns of the action potential are played back into each neuron using the voltage clamp. The participant presses a button during the replay because the relevant motor neurons are activated. **(D)** Activity in 2 representative neurons from the participant’s brain; during the recording, neuron ***a*** fires at time *T*_1_ and causes action potential in neuron ***b*** at time *T*_2_. Red arrows indicate the direction of cause and effect between the neurons and the controller; Neuron ***a*** causes action potential in neuron ***b***, and both are recorded by the controller. **E**. During replay, neuron ***a*** and neuron ***b*** fire exactly at times *T*_1_ and *T*_2_, respectively, as in C, but both action potentials are caused by the replay controller (depicted by the green arrows). In both C and D, the action potentials propagate through the axon (depicted by the red arrow), but in D, they do not affect the firing of neuron *b*.

Here, we revisited “The Story of a Brain” by Zuboff [36] in light of the advances in neurotechnology and their potential role in unraveling the neural causes of consciousness. We consider the consequences of an experiment where a participant’s brain is manipulated in 3 steps using extrapolated versions of technologies currently within the grasp of neuroscience [37], specifically, voltage clamp and optogenetics. Voltage clamp [38,39] can fully and precisely determine (i.e., clamp) the neuron’s membrane potential. An amplifier computes the current to be injected into a neuron via an electrode such that the neuron voltage matches the experimenter’s “command” voltage. An intelligent offspring of the voltage clamp is the “action potential clamp” [40], which, as its name suggests, clamps the neuron voltage to a previously recorded action potential (**Fig 1A** and see [41,42]). The replay, which voltage clamps the neurons, is not simply superimposed on the neuron’s activity but rather fully determines it by overriding all naturally occurring voltage changes. Consequently, the role of connectivity, feedback connections [43,44], and information propagation is subsumed by the replay.

For each step in the thought experiment, we ask whether activating the brain with an artificial replay of previously recorded action potentials would result in conscious perception and explore the possible outcomes. It is impossible to say whether replaying and recording all the neurons in the entire brain will be feasible in the future. However, resources from funding agencies (e.g., The BRAIN Initiative, the SIMONS foundation, and others), large-scale research projects (such as the Human Brain Project, The Connectome Project, and the Brain Activity Map Project), and the barrage of new studies and new technologies mentioned above [22,23] show the implicit (if not explicit) steps toward this goal. As the community has decided to step in this direction and prioritize the development of “large-scale monitoring” and “precise interventional tools” (BRAIN Initiative recommendations for 2025), we should consider the consequences of this endeavor for solving the fundamental problem of consciousness if/when it is successful.

Our immediate aim is to challenge the primacy of action potentials as an explanation for consciousness. Action potentials are the brain’s main signaling mechanisms, and they form the basis for neural computation as we understand it. But our broader goal is to clarify the limitations of measured neural biological and electrical properties in laboratory settings to explain consciousness.

### Recording and controlling consciousness in 3 experimental steps

We start with the working hypothesis that consciousness is caused by the neuronal firing of action potentials in the brain. We will ask the reader to either accept or reject the working hypothesis after each of 3 successive manipulations (steps) of a participant’s brain. Initially (**Fig 1B**), we record all action potentials from all the neurons in an awake participant’s brain while she is presented with a green light (see **Discussion** for generalization for other neuronal properties). The participant presses a button to report that she perceives the green light consciously. This kind of experiment, where a participant reports a simple stimulus perception, is typical for studying consciousness [30,32]. The minimalistic experimental setting (i.e., seeing green light and pressing a button) captures the essence and avoids distractions. Such as emotional responses or free will that may appear in more complex experiments. Furthermore, the simple experiment could be generalized to more complex real-life–like conditions (e.g., watching a movie) without affecting the conclusion.

#### Step 1: Removing cause-and-effect relations between the neurons

Next, we force all of the participant’s neurons to fire by playing back the trains of action potentials recorded previously during the conscious perception task (in **Fig 1C and 1E**). For the replay, we voltage clamp the cell bodies of all the neurons. The purpose of the voltage clamp is 2-fold: to force the membrane potential to be identical to the recorded potential and to override any other input that would otherwise influence the neuron. The motor neurons that caused the participant to voluntarily push the button (**Fig 1B and 1D**) are now activated by a replay controller, and, therefore, the participant pushes the button (**Fig 1C and 1E**). Moreover, by pushing the button, the participant (seemingly) reports her conscious perception of green light since the neurons that control motor output are also forced to fire as before. But does the participant really experience the green light during the replay?

Answering “no” entails the rejection of the working hypothesis because it implies that something other than the action potentials is responsible for the conscious perception of green light. Rejecting the hypothesis challenges widely held intuitions in neuroscience, namely, the centrality of brain activity in the form of firing neurons to consciousness. The problem with arguing that the participant is unconscious in this step is that identical brain activities (**Fig 1B** versus **Fig 1C**) result in different outcomes; consciously perceiving green during the recording versus being unconscious during the replay (see **Discussion**). Alternatively, answering “yes” (i.e., that the participant has conscious experience of green light) takes us to the next step.

#### Step 2: Optogenetically disconnecting the neurons

**Fig 2:**
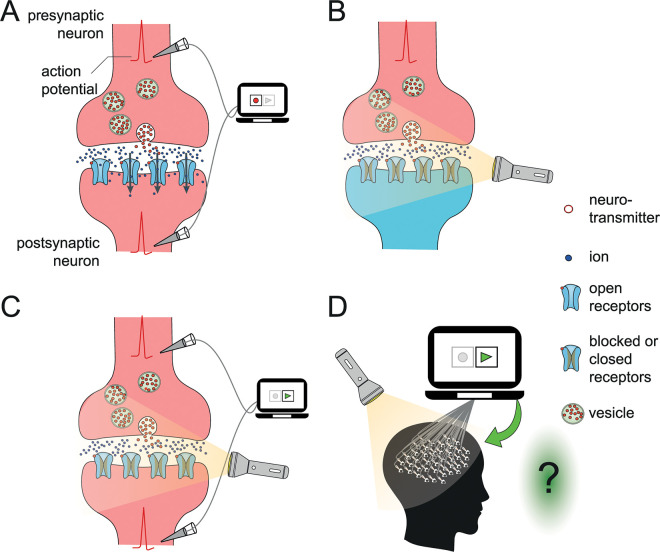
Step 2: Blocking all synaptic transmission in the brain. Normal postsynaptic channel receptors were replaced by light-sensitive (but otherwise identical) postsynaptic channel receptors. Thus, we could use light to block the synaptic transmission optogenetically and, therefore, reversibly disconnect all the neurons from each other. **(A)** The diagram shows the recording of action potentials (in Step 1) from the pre- and postsynaptic neurons. An action potential at the synaptic terminal of the presynaptic neuron causes vesicles to release neurotransmitters. **(B)** In Step 2, the permeability of the genetically modified postsynaptic channel receptors is blocked using light. Consequently, action potentials in the presynaptic neurons cannot influence the generation of action potentials in postsynaptic neurons (even when the neurotransmitter binds to the channel receptors). **(C, D)** The light-induced synaptic disconnection is bypassed when the action potentials recorded in Step 1 are played back into both the pre- and postsynaptic neurons (C) for all the neurons in the participant’s brain (D).

In this step, we use optogenetic tools to disconnect all the synapses in the participant’s brain (for details, see **Fig 2**). By illuminating our participant’s brain, we block synaptic transmission and functionally disconnect all the neurons from each other. Switching off the light releases the block and causes the synapses to reconnect. Typically, blocking synaptic transmission in the brain will dramatically change the neurons’ firing patterns by preventing neurons from activating each other. However, because we control the firing of all neurons, they fire precisely as in Step 1, despite being disconnected from each other. Consequently, the brain activates the motoneurons in the spinal cord (these connections were not optogenetically blocked), and the participant presses the button, seemingly informing us that she is conscious of the green light. As in the previous step, we ask the reader to evaluate whether the participant consciously perceived green light during the replay, although all neurons are disconnected.

Answering “no” at this step implies that, although the artificial replay leaves consciousness intact (i.e., “yes” in Step 1), manipulation of the synapses that bears no consequences on the generation of these action potentials (in both Step 1 and Step 2) eliminates conscious perception. In other words, answering “no” is to reject the working hypothesis and to suggest that biochemical processes at the synaptic site play a central role in consciousness (see **Discussion** for generalization beyond action potentials). Alternatively, answering “yes” takes us to the next step.

#### Step 3: Physically disconnecting the neurons

**Fig 3:**
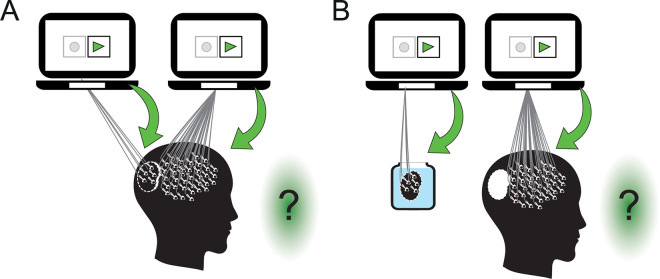
Step 3: Surgically removing brain tissue. **(A, B)** The visual cortex is resected and then the action potentials are played back, respectively, to the removed visual cortex and the rest of the intact brain. We can either leave the resected visual cortex in its place (A) or remove it from the participant’s brain (B).

Temporal lobe seizures, a common type of epilepsy, are often treated with resective surgery in patients that show resistance to drug therapy. A large section of the temporal cortex (considered healthy) is surgically removed to access the deeper brain regions containing the focus of epilepsy. Remarkably, many of the neurons can be kept alive and well for a couple of days after the surgery [45] (see also [46]), and, therefore, they are often used for experimentation [15] (e.g., [47]). Rather than the temporal cortex, in this step, we surgically cut (**Fig 3A**) and remove (**Fig 3B)** the visual cortex from the participant’s brain. In contrast to today’s surgical methods, we require a more subtle approach that keeps the resected tissue largely undamaged. We play the action potentials back into all the neurons, including the neurons in the resected areas, first after cutting (**Fig 3A**) and then after removing them from the rest of the brain (**Fig 3B**). Will the participant consciously perceive green light during the replay, despite resecting the region responsible for the perception of vision and color?

Answering “no” after the resective surgery (**Fig 3A and 3B**) challenges the reader to explain why, although the synaptic disconnection at a molecular scale in Step 2 (**Fig 2**) does not change the conscious perception, the physical disconnection with a surgical scalpel nevertheless changes the participant’s conscious perception. Answering “yes” after surgically cutting the visual cortex (**Fig 3A**) but “no” after its removal (**Fig 3B**) implies that the distance of the resected neurons from the rest of the brain is vital for conscious perception. The distinction between surgery with (**Fig 3A**) and without the removal (**Fig 3B**) of the visual cortex raises interesting questions regarding the effect of the distance between brain regions on consciousness. For example, does the brain’s size (between species and even within the same species) affect consciousness due to the distance between brain regions?

If the reader answers “yes” in Step 3, then a second resection or any number of additional resections should not change the reader’s answer. Iteratively resecting and re-resecting eventually leaves us with a brain in the form of geographically scattered individual neurons. Therefore, accepting the hypothesis in Step 3 results in a conscious scattered brain. The alternative, namely, arguing that scattered brains cannot be conscious, leads to rejecting the hypothesis that the firing of the neurons causes our conscious experience.

**Fig 4:**
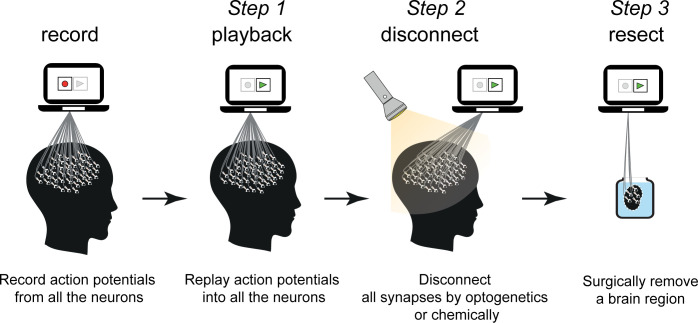
Summary of the experiment. Recording, replaying (Step 1), disconnecting all neurons in the brain (Step 2) and surgically removing the visual cortex (Step 3). After each step where the participant’s brain was manipulated, the reader is asked to evaluate whether or not the participant still has the conscious perception of the green light.

In 3 progressing steps, we manipulated our participant’s brain (**Fig 4**) and tested the hypothesis that the neuronal processes in our brain cause conscious perception. At first, the experiment presented here might appear similar to a thought experiment described by Zenon Pylyshyn [48], where neurons were gradually replaced by microchips with identical functionality. However, Pylyshyn aimed to preserve the cause-and-effect relations between the neurons while eliminating the biological substrate, whereas here the biological substrate was preserved (at least in the first 2 steps) while eliminating the cause and effect between the neurons.

### An overview and discussion of the experimental steps

Initially, we recorded the neuronal firing in the entire brain of a participant while she was consciously experiencing green light. In Step 1, we played the recorded action potentials back to each neuron in the participant’s brain (replay), artificially recreating the brain activity that was naturally caused by the visual stimulus. It is important to note that, although the replay was artificial in our experiment, it is a known biological process thought to underlie perception, memory consolidation, and network homeostasis in animals [49] and humans [50–52].

Because the effect of synaptic connections on brain activity was already precluded in Step 1 by imposing the replay, disconnecting the synapses either optogenetically or physically (in Step 2 and Step 3, respectively) has no further consequences on the firing of action potentials. Therefore, if the reader does not reject the hypothesis at the first step, why reject it in further steps (see further discussion in theories of consciousness)? Finally, we argued that if the participant consciously perceived the green light after the resective surgery (Step 3), it would imply that a scattered brain can be conscious.

In the working hypothesis, we only considered whether action potentials cause consciousness. Performing our experiments for other neuronal processes might be more difficult than for action potentials and, in some cases, even impossible. However, conceptually, it is straightforward to include them in the hypothesis and even include combinations of multiple processes; for example, membrane potential fluctuations, calcium ion concentrations [53,54], the release of neurotransmitters from the presynaptic terminals, or activity in glial cells [55,56]. To consider multiple biological processes, we first need to record these processes and then test the hypothesis against Steps 1 to 3 by asking in each step whether the participant’s conscious perception changed when the respective cellular processes remained exactly the same.

Bayne and colleagues [57] discuss the circumstances, plausibility, and consequences of “islands of awareness” occurring in brains that cannot interact with the natural world via perception and action. Interestingly, Bayne and colleagues argue that islands of awareness can also exist when the brain is physically isolated from a body. Such cases are ex cranio brains, disconnected hemispheres post-hemispherectomy, and cerebral organoids. It is worth noting that the brain during the replay is fundamentally different from such islands of awareness because it effectively contains both the input and the output. The stimulus triggered the trains of action potentials that constitute the replay. The replayed action potentials activate the spinal motoneurons causing the participant to press the button. Although it is difficult to assess whether the brain is conscious during the replay, it is not isolated from the world.

### Implications for theories of consciousness

The experiment we described here is useful as a benchmark for theories of consciousness, revealing hidden incoherences and ambiguities [58]. Specifically, for a given theory of consciousness, we ask in which step (i.e., Steps 1 to 3) and why we would reject the working hypothesis and claim that the participant loses consciousness.

By our estimation, several theories of consciousness would predict that the participant is conscious after all the manipulations from Step 1 to Step 3. In particular, theories that specify the neurobiological mechanisms for consciousness in detail—unless they make some additional assumptions—are forced to conclude that scattered brains are conscious. If conscious experience is caused by action potentials fired by particular neurons, as in the theory by Crick and Koch [25,59], why should disconnecting these neurons or scattering them affect consciousness? Even considering further details of this theory, e.g., that the brainstem and higher-order thalamic nuclei have an enabling role in consciousness [59,60], the theory does not conflict with a conscious participant throughout Steps 1 to 3.

Recurrent processing (RP) theory by Lamme [61,62] relates consciousness to feedback between different cortical areas. The feedback is essentially the influence of some neurons, causing action potentials in other neurons. The firing caused by the feedback could just as well be replayed even in disconnected and scattered scenarios. Again, if the role of feedback is to cause action potentials in certain neurons, then nothing in this theory suggests a loss of consciousness in Steps 1 to 3.

We recently proposed the dendritic integration theory [DIT; 63,64], which hypothesizes that consciousness depends on the reintegration of top-down information via the apical dendrites of layer 5 pyramidal neurons. DIT is based on the empirical finding that the electrical coupling between apical and basal dendrites of cortical pyramidal neurons is disrupted by common anesthetics, thus blocking the influence of the apical dendrite on the output of the layer 5 pyramidal neurons [65]. According to this theory, decoupling the apical from the basal dendrites in a sufficiently large number of cortical pyramidal neurons would switch off consciousness. Essentially, DIT pinpoints the nexus of information flow within the brain microarchitecture that is crucial for consciousness. Besides the clinical benefit and understanding of the computation involved, DIT offers a framework for interrogating this biological mechanism in the laboratory.

Regarding the thought experiment presented here, however, placing an electrode at the cell body to generate the cellular output effectively bypasses the critical nexus point in the apical dendrite. We, therefore, predict that the replay of activity at the cell bodies of pyramidal cells would, in this case, completely entail the former influence of the apical dendrite. Furthermore, DIT is agnostic about the intrinsic necessity of apical causality, per se, versus the resultant firing activity at the cell body. In this respect, DIT does not inform us whether the brain is conscious under replay or whether scattered brains are conscious.

A similar conclusion is implied by functionalist theories, which do not commit to a particular neural implementation, but rather suggest that consciousness is related to specific functions or processes regardless of the exact implementation. For instance, the global neuronal workspace (GNW) theory [66–68] suggests that “global broadcasting constitutes the physiological correlate of conscious reportability” [69]. Similarly, the higher-order thought (HOT) theory of consciousness is a functionalist theory that relates consciousness to metacognitive higher-order processing [70,71]. As “global broadcasting” and higher-order processing are functions that are not restricted to brains [72,73], these theories do not necessarily conflict with the notion that the replay generates consciousness (Step 1). In particular, the same patterns of global broadcasting, self-sustained activity and ignition dynamics central to the GNW [66,68], and the higher-order processing central to HOT [70,71] could be exactly replayed in Step 1. The proponents of these theories might be more skeptical of Step 2 and Step 3, but nothing inherent to these theories would prohibit a disconnected or scattered brain from being conscious.

According to Zeki and colleagues [74], consciousness comprises nodes of micro-consciousnesses in different brain regions. Interestingly, in contrast to other theories, Zeki argues that consciousness is not unified [75]. Therefore, color and motion, for example, are consciously perceived in different parts of the cortex and only then bind together with other nodes to form a macro-consciousness. A micro-consciousness is autonomous [76] and does not require further processing. Therefore, the resected visual cortex in Step 3 may become micro-conscious of the green light during the replay. We could not find a direct reason as to why, according to Zeki, scattered brains during replay cannot bind together into a macro-consciousness.

In conclusion, some theories do not have conceptual reasons or assumptions as to why replaying, disconnecting, or scattering the neurons should lead to a loss of consciousness. What are the additional assumptions needed to escape these conclusions? Essentially there are 2 possibilities: one, that the three-dimensional structure of the brain is necessary for consciousness, and the other that the intrinsic cause and effect (i.e., between the neurons) is necessary (see the “The replay conundrum” section). Even if these theories make one of these additional assumptions, what is the justification? Is it only an ad hoc assumption to avoid the conclusion that scattered brains could be conscious?

#### Consciousness is lost in Step 3: Theories that require the structure of the brain

The brain’s particular three-dimensional structure is central for theories that associate consciousness with the electromagnetic field [77–83]. These theories would predict that the participant consciously experiences green light as long as the electrical field remains as it was during the recording. Our participant, therefore, will remain conscious of the green light during the replay (Step 1) and after synaptic disconnection (Step 2*)*, given that these steps do not interfere with the brain’s field relevant to consciousness. However, the participant’s conscious experience may change by surgically removing the visual cortex (Step 3), altering the brain’s physical structure and, consequently, the electromagnetic field. This offers an explanation as to why a scattered brain cannot be conscious.

#### Consciousness is lost in Step 1: Theories that require intrinsic cause and effect

Integrated information theory [84–86] quantifies consciousness based on the repertoire of all possible cause-and-effect interactions between the neurons in the brain’s network. Disconnecting the neurons in Step 2 abolished the network structure that underlies the interaction between neurons. However, in Step 1, the replay imposed particular (recorded) trains of action potentials and effectively vetoed all the interaction between the neurons, even though the synaptic connections were fully functional. Therefore, according to the assumptions of IIT, our participant already loses consciousness in Step 1.

According to Searle’s “biological naturalism” [87] (which is an approach rather than a theory that does not specify the biological mechanism for consciousness), the replay in Step 1 does not necessarily result in loss of consciousness. The participant will remain conscious during the replay as long as the underlying biological substrate and the “right” causal powers are intact. Therefore, according to Searle, it is not evident if and in which precise step loss of consciousness would happen. The right causal powers may lie, for example, in the propagation of the action potentials within the axon. In this case, our participant would remain conscious during the replay because both the naturally occurring and the artificially induced action potentials propagate via the axon. According to Searle, replaying other biological processes may have different outcomes. Therefore, the replay could be insightful in revealing the particular causal powers that matter for consciousness.

### The replay conundrum

To underscore the usefulness of replay as a potential experimental strategy, let us compare the replay of brain activity to a detailed simulation of the brain. A frequent objection to the view that a detailed simulation of the human brain can become conscious is that it merely manipulates symbols whose meaning depends on external interpretation, whereas neural activity is intrinsically meaningful to the brain [88]. In contrast to a simulation, the artificial neuronal firing induced by the replay is intrinsically meaningful to the brain/participant because it is an identical copy of intrinsically meaningful activity (i.e., an experience of green light). John Searle famously explained that “you could not digest pizza by running the program that simulates such digestion” [89]. Unlike biologically detailed simulations running on a computer, the replay is recorded and activated on the same substrate. Therefore, in contrast to a simulation of the stomach, recording and then replaying smooth muscle contraction and enzyme secretion would result in digestion. What would it imply about the nature of consciousness if replay would work for stomach digestion or the heart pumping blood but not for the brain and consciousness?

Several theories of consciousness claim that cause-and-effect relations among the neurons within the brain are decisive for consciousness, either with or without strictly insisting on the necessity of the biological substrate. For some theories, cause and effect between the neurons is no more than a mechanism responsible for generating patterns of brain activity. For other theories, cause and effect is more fundamental, and without it, consciousness cannot exist (for extended discussion, see the previous section). Because the replay abolishes the biological intrinsic cause-and-effect relations between the neurons, the later theories assert that our participant loses consciousness during the replay. However, the analogous assertion concerning the heart and stomach does not work; even without cause and effect between the elements of the heart, replaying myocardial cells’ activity would do the biological and mechanical work of pumping blood. Why should a replay of action potentials in the case of the brain and consciousness not have a similar outcome to the case of stomach and food digestion or heart and pumping blood? Possibly, cause and effect between the neurons is required only in the case of the brain and consciousness. The replay could be viewed as analogous to brain lesion experiments, but rather than removing a piece of tissue, we remove cause and effect to examine whether the basis for consciousness is either neuronal activity or cause and effect (or both). Our experiment is a plausible and decisive strategy to distinguish between these 2 possibilities.

### The replay’s practical implications

Whether or not the participant loses consciousness during the replay has concrete, practical consequences. For some theories, experiments using whole-brain replay [90] (such as the study of the neuronal microcircuitry of agonizing pain) would be ethically unacceptable without proper animal welfare measures because the animals will consciously experience the effects of replay during the experiment. In contrast, for other theories, a whole-brain replay may reduce the ethical concerns to a minimum because it is identical to potent anesthesia and a complete loss of consciousness but allows studying the active brain. An animal that expresses agony during such an experiment is similar to the unconscious participant pushing the button during the replay, i.e., it would not feel anything consciously.

Neural prosthetics and particularly visual prosthetics may provide clues as to whether activating the brain by replay results in loss of consciousness. Blind patients can see with implants of an electrode array in their visual cortex [91]. Neurons activated by electrodes in the visual cortex generate visual perception and even color perception [92,93]. However, the brain–machine interfaces are currently limited in the number of neurons they can precisely stimulate. According to some theories we mentioned in the previous section, a naive attempt to improve vision quality by increasing the size of the stimulated brain area and the number of stimulating electrodes (i.e., use the replay with more neurons) could paradoxically diminish the conscious visual experience rather than enhance it. According to the view that cause and effect is crucial for consciousness, there is a theoretical upper limit to the size of the brain area one can stimulate. Above this limit, the stimulation may prohibit neurons from affecting one another and curtail conscious experience.

## Concluding remarks

Does the replay of recorded action potentials to the entire brain result in the loss of consciousness? And if so, what are the implications for our ability to study consciousness on the basis of neural activity? Would the right technology make these questions a matter of experimental investigation rather than (or in addition to) a philosophical debate? Towards answering these questions, our thought experiment makes an important step towards challenging the conventional wisdom regarding the causal link between action potentials and consciousness.

It should be noted that the scope of this experiment is not restricted to brains. It may apply to nonbiological substrates one might suspect to be conscious such as computer hardware [94] and software [69,95], where recording and replaying every aspect of the activity and information flow are almost unconstrained. The implications of this thought experiment, therefore, extend to questions about artificial intelligence and consciousness. So, to end where we started, do action potentials cause consciousness? As our understanding of the brain progresses, we will inevitably be confronted with this seemingly simple question. The thought experiment we presented here demonstrated that even with advanced technologies, the answer might be less obvious than we think.

## Abbreviations

DIT: dendritic integration theory
GNW: global neuronal workspace
HOT: higher-order thought
RP: recurrent processing

## References

[1] Brindley GS, Lewin WS. The sensations produced by electrical stimulation of the visual cortex. J Physiol. 1968;196:479–93. doi: 10.1113/jphysiol.1968.sp008519 4871047 PMC1351724

[2] Foerster O. Beitrage zur Pathophysiologie der Sehbahn und der Sehsphare. J Psychol Neurol Lpz. 1929;39:463–85.

[3] Krause F, Schum H. Die Epileptischen Erkrankungen. In: Kuttner H, editor. Neue Deutsche Chirurgie. Stuttgart: Enke; 1931. pp. 482–486.

[4] Penfield W, Perot P. The brain’s record of auditory and visual experiencea final summary and discussion. Brain. 1963;86:595–696. doi: 10.1093/brain/86.4.595 14090522

[5] Lebedev MA, Nicolelis MAL. Brain-Machine Interfaces: From Basic Science to Neuroprostheses and Neurorehabilitation. Physiol Rev. 2017;97:767–837. doi: 10.1152/physrev.00027.2016 28275048

[6] O’Doherty JE, Lebedev MA, Ifft PJ, Zhuang KZ, Shokur S, Bleuler H, et al. Active tactile exploration enabled by a brain-machine-brain interface. Nature. 2011;479:228–31. doi: 10.1038/nature10489 21976021 PMC3236080

[7] Zanos S, Richardson AG, Shupe L, Miles FP, Fetz EE. The Neurochip-2: An Autonomous Head-Fixed Computer for Recording and Stimulating in Freely Behaving Monkeys. IEEE Trans Neural Syst Rehabil Eng Publ IEEE Eng Med Biol Soc. 2011;19:427–35. doi: 10.1109/TNSRE.2011.2158007 21632309 PMC3159515

[8] Zhou A, Santacruz SR, Johnson BC, Alexandrov G, Moin A, Burghardt FL, et al. A wireless and artefact-free 128-channel neuromodulation device for closed-loop stimulation and recording in non-human primates. Nat Biomed Eng. 2019;3:15–26. doi: 10.1038/s41551-018-0323-x 30932068

[9] Lebedev MA, Nicolelis MAL. Chapter 3—Toward a whole-body neuroprosthetic. In: Schouenborg J, Garwicz M, Danielsen N, editors. Progress in Brain Research. Elsevier; 2011. pp. 47–60. doi: 10.1016/B978-0-444-53815-4.00018-2 21867793

[10] Beauchamp MS, Oswalt D, Sun P, Foster BL, Magnotti JF, Niketeghad S, et al. Dynamic Stimulation of Visual Cortex Produces Form Vision in Sighted and Blind Humans. Cell. 2020;181:774–783.e5. doi: 10.1016/j.cell.2020.04.033 32413298 PMC7331799

[11] Kato K, Sawada M, Nishimura Y. Bypassing stroke-damaged neural pathways via a neural interface induces targeted cortical adaptation. Nat Commun. 2019;10:4699. doi: 10.1038/s41467-019-12647-y 31619680 PMC6796004

[12] Lacagnina AF, Brockway ET, Crovetti CR, Shue F, McCarty MJ, Sattler KP, et al. Distinct hippocampal engrams control extinction and relapse of fear memory. Nat Neurosci. 2019;22:753–61. doi: 10.1038/s41593-019-0361-z 30936555 PMC6705137

[13] Liu X, Ramirez S, Pang PT, Puryear CB, Govindarajan A, Deisseroth K, et al. Optogenetic stimulation of a hippocampal engram activates fear memory recall. Nature. 2012;484:381–5. doi: 10.1038/nature11028 22441246 PMC3331914

[14] Josselyn SA, Tonegawa S. Memory engrams: Recalling the past and imagining the future. Science. 2020;367. doi: 10.1126/science.aaw4325 31896692 PMC7577560

[15] Peng Y, Mittermaier FX, Planert H, Schneider UC, Alle H, Geiger JRP. High-throughput microcircuit analysis of individual human brains through next-generation multineuron patch-clamp. Huguenard J, Marder E, Jarsky T, editors. Elife. 2019;8: e48178. doi: 10.7554/eLife.48178 31742558 PMC6894931

[16] Reyes AD. Synchrony-dependent propagation of firing rate in iteratively constructed networks in vitro. Nat Neurosci. 2003;6:593–9. doi: 10.1038/nn1056 12730700

[17] Chung JE, Joo HR, Fan JL, Liu DF, Barnett AH, Chen S, et al. High-Density, Long-Lasting, and Multi-region Electrophysiological Recordings Using Polymer Electrode Arrays. Neuron. 2019;101:21–31.e5. doi: 10.1016/j.neuron.2018.11.002 30502044 PMC6326834

[18] Kauvar IV, Machado TA, Yuen E, Kochalka J, Choi M, Allen WE, et al. Cortical Observation by Synchronous Multifocal Optical Sampling Reveals Widespread Population Encoding of Actions. Neuron 2020;0. doi: 10.1016/j.neuron.2020.04.023 32433908 PMC7687350

[19] Kim TH, Zhang Y, Lecoq J, Jung JC, Li J, Zeng H, et al. Long-Term Optical Access to an Estimated One Million Neurons in the Live Mouse Cortex. Cell Rep. 2016;17:3385–94. doi: 10.1016/j.celrep.2016.12.004 28009304 PMC5459490

[20] Steinmetz NA, Koch C, Harris KD, Carandini M. Challenges and opportunities for large-scale electrophysiology with Neuropixels probes. Curr Opin Neurobiol. 2018;50:92–100. doi: 10.1016/j.conb.2018.01.009 29444488 PMC5999351

[21] Stirman JN, Smith IT, Kudenov MW, Smith SL. Wide field-of-view, multi-region, two-photon imaging of neuronal activity in the mammalian brain. Nat Biotechnol. 2016;34:857–62. doi: 10.1038/nbt.3594 27347754 PMC4980167

[22] Clough M, Chen JL. Cellular resolution imaging of neuronal activity across space and time in the mammalian brain. Curr Opin Biomed Eng. 2019;12:95–101. doi: 10.1016/j.cobme.2019.11.004 32104747 PMC7043406

[23] Kleinfeld D, Luan L, Mitra PP, Robinson JT, Sarpeshkar R, Shepard K, et al. Can One Concurrently Record Electrical Spikes from Every Neuron in a Mammalian Brain? Neuron. 2019;103:1005–15. doi: 10.1016/j.neuron.2019.08.011 31495645 PMC6763354

[24] Sahasrabuddhe K, Khan AA, Singh AP, Stern TM, Ng Y, Tadić A, et al. The Argo: A 65,536 channel recording system for high density neural recording in vivo. bioRxiv. 2020:2020.07.17.209403. doi: 10.1101/2020.07.17.209403

[25] Crick F, Koch C. Towards a neurobiological theory of consciousness. Semin Neurosci. 1990;2:263–75.

[26] Crick F, Koch C. Consciousness and neuroscience. Cereb Cortex. 1998;8:97–107. doi: 10.1093/cercor/8.2.97 9542889

[27] Searle JR. How to Study Consciousness Scientifically. Philos Trans Biol Sci. 1998;353:1935–42. 10.1098/rstb.1998.0346 PMC1692422 9854266

[28] Tononi G. An information integration theory of consciousness. BMC Neurosci. 2004;5:42. doi: 10.1186/1471-2202-5-42 15522121 PMC543470

[29] Tononi G. Edelman, Gerald M. Consciousness and Complexity. Science. 1998;282:1846–51. doi: 10.1126/science.282.5395.1846 9836628

[30] Aru J, Bachmann T, Singer W, Melloni L. Distilling the neural correlates of consciousness. Neurosci Biobehav Rev. 2012;36:737–46. doi: 10.1016/j.neubiorev.2011.12.003 22192881

[31] de Graaf TA, Hsieh P-J, Sack AT. The ‘correlates’ in neural correlates of consciousness. Neurosci Biobehav Rev. 2012;36:191–7. doi: 10.1016/j.neubiorev.2011.05.012 21651927

[32] Rees G, Frith C, Lavie N. Processing of irrelevant visual motion during performance of an auditory attention task. Neuropsychologia. 2001;39:937–49. doi: 10.1016/s0028-3932(01)00016-1 11516446

[33] Casali AG, Gosseries O, Rosanova M, Boly M, Sarasso S, Casali KR, et al. A Theoretically Based Index of Consciousness Independent of Sensory Processing and Behavior. Sci Transl Med. 2013;5:198ra105–198ra105. doi: 10.1126/scitranslmed.3006294 23946194

[34] Monti MM, Vanhaudenhuyse A, Coleman MR, Boly M, Pickard JD, Tshibanda L, et al. Willful Modulation of Brain Activity in Disorders of Consciousness. N Engl J Med. 2010;362:579–89. doi: 10.1056/NEJMoa0905370 20130250

[35] Owen AM, Coleman MR, Boly M, Davis MH, Laureys S, Pickard JD. Detecting Awareness in the Vegetative State. Science. 2006;313:1402–2. doi: 10.1126/science.1130197 16959998

[36] Zuboff A. The Story of a Brain. 1981. pp. 202–212.

[37] Roelfsema PR, Denys D, Klink PC. Mind Reading and Writing: The Future of Neurotechnology. Trends Cogn Sci. 2018;22:598–610. doi: 10.1016/j.tics.2018.04.001 29729902

[38] Cole KS. Dynamic electrical characteristics of the squid axon membrane. Arch Sci Physiol (Paris). 1949;3:253–8.

[39] Cole KS. Membranes, Ions and Impulses: A Chapter of Classical Biophysics. University of California Press. 1972.

[40] Starzak ME, Starzak RJ. An Action Potential Clamp to Probe the Effectiveness of Space Clamp in Axons. IEEE Trans Biomed Eng. 1978;BME-25:201–4. doi: 10.1109/TBME.1978.326249 640708

[41] Llinás R, Joyner RW, Nicholson C. Equilibrium Potential for the Postsynaptic Response in the Squid Giant Synapse. J Gen Physiol. 1974;64:519–35. doi: 10.1085/jgp.64.5.519 4374500 PMC2226163

[42] Llinás R, Sugimori M, Simon SM. Transmission by presynaptic spike-like depolarization in the squid giant synapse. Proc Natl Acad Sci U S A. 1982;79:2415–9. doi: 10.1073/pnas.79.7.2415 6954549 PMC346205

[43] Lamme VAF, Zipser K, Spekreijse H. Figure-ground activity in primary visual cortex is suppressed by anesthesia. Proc Natl Acad Sci U S A 1998;95:3263–8. doi: 10.1073/pnas.95.6.3263 9501251 PMC19730

[44] Supèr H, Lamme VAF. Altered figure-ground perception in monkeys with an extra-striate lesion. Neuropsychologia. 2007;45:3329–34. doi: 10.1016/j.neuropsychologia.2007.07.001 17692346

[45] Wickham J, Brödjegård NG, Vighagen R, Pinborg LH, Bengzon J, Woldbye DPD, et al. Prolonged life of human acute hippocampal slices from temporal lobe epilepsy surgery. Sci Rep. 2018;8:4158. doi: 10.1038/s41598-018-22554-9 29515159 PMC5841387

[46] Vrselja Z, Daniele SG, Silbereis J, Talpo F, Morozov YM, Sousa AMM, et al. Restoration of brain circulation and cellular functions hours post-mortem. Nature. 2019;568:336–43. doi: 10.1038/s41586-019-1099-1 30996318 PMC6844189

[47] Gidon A, Zolnik TA, Fidzinski P, Bolduan F, Papoutsi A, Poirazi P, et al. Dendritic action potentials and computation in human layer 2/3 cortical neurons. Science. 2020;367:83–7. doi: 10.1126/science.aax6239 31896716

[48] Pylyshyn ZW. The ‘causal power’ of machines. Behav Brain Sci. 1980;3:442–4. doi: 10.1017/S0140525X0000594X

[49] Liu T-Y, Watson BO. Patterned activation of action potential patterns during offline states in the neocortex: replay and non-replay. Philos Trans R Soc B Biol Sci. 2020;375:20190233. doi: 10.1098/rstb.2019.0233 32248782 PMC7209911

[50] Liu Y, Dolan RJ, Kurth-Nelson Z, Behrens TEJ. Human Replay Spontaneously Reorganizes Experience. Cell. 2019;178:640–652.e14. doi: 10.1016/j.cell.2019.06.012 31280961 PMC6657653

[51] Schuck NW, Niv Y. Sequential replay of nonspatial task states in the human hippocampus. Science. 2019;364. doi: 10.1126/science.aaw5181 31249030 PMC7241311

[52] Vaz AP, Wittig JH, Inati SK, Zaghloul KA. Replay of cortical spiking sequences during human memory retrieval. Science. 2020;367:1131–4. doi: 10.1126/science.aba0672 32139543 PMC7211396

[53] Ma G, Wen S, He L, Huang Y, Wang Y, Zhou Y. Optogenetic toolkit for precise control of calcium signaling. Cell Calcium. 2017;64:36–46. doi: 10.1016/j.ceca.2017.01.004 28104276 PMC5457325

[54] Ziv Y, Ghosh KK. Miniature microscopes for large-scale imaging of neuronal activity in freely behaving rodents. Curr Opin Neurobiol. 2015;32:141–7. doi: 10.1016/j.conb.2015.04.001 25951292

[55] Rost BR, Schneider F, Grauel MK, Wozny C, Bentz C, Blessing A, et al. Optogenetic Acidification of Synaptic Vesicles and Lysosomes. Nat Neurosci. 2015;18:1845–52. doi: 10.1038/nn.4161 26551543 PMC4869830

[56] Rost BR, Schneider-Warme F, Schmitz D, Hegemann P. Optogenetic Tools for Subcellular Applications in Neuroscience. Neuron. 2017;96:572–603. doi: 10.1016/j.neuron.2017.09.047 29096074

[57] Bayne T, Seth AK, Massimini M. Are There Islands of Awareness? Trends Neurosci. 2020;43:6–16. doi: 10.1016/j.tins.2019.11.003 31836316

[58] Kuhn T. A Function for Thought Experiments. The Essential Tension: Selected Studies in Scientific Tradition and Change. University of Chicago Press; 1964. pp. 240–265.

[59] Crick F, Koch C. A framework for consciousness. Nat Neurosci. 2003;6:119–26. doi: 10.1038/nn0203-119 12555104

[60] Koch C. The Quest for Consciousness: A Neurobiological Approach. 1st Edition. Denver, Colo.: W. H. Freeman; 2004.

[61] Lamme VAF. Why visual attention and awareness are different. Trends Cogn Sci. 2003;7:12–8. doi: 10.1016/s1364-6613(02)00013-x 12517353

[62] Lamme VAF. Separate neural definitions of visual consciousness and visual attention; a case for phenomenal awareness. Neural Netw. 2004;17:861–72. doi: 10.1016/j.neunet.2004.02.005 15288903

[63] Aru J, Suzuki M, Larkum ME. Cellular Mechanisms of Conscious Processing. Trends Cogn Sci. 2020;24:814–25. doi: 10.1016/j.tics.2020.07.006 32855048

[64] Larkum M. A cellular mechanism for cortical associations: an organizing principle for the cerebral cortex. Trends Neurosci. 2013;36:141–51. doi: 10.1016/j.tins.2012.11.006 23273272

[65] Suzuki M, Larkum ME. General Anesthesia Decouples Cortical Pyramidal Neurons. Cell. 2020;180:666–676.e13. doi: 10.1016/j.cell.2020.01.024 32084339

[66] Dehaene S, Changeux J-P. Experimental and Theoretical Approaches to Conscious Processing. Neuron. 2011;70:200–27. doi: 10.1016/j.neuron.2011.03.018 21521609

[67] Dehaene S, Naccache L. Towards a cognitive neuroscience of consciousness: basic evidence and a workspace framework. Cognition. 2001;79:1–37. doi: 10.1016/s0010-0277(00)00123-2 11164022

[68] Mashour GA, Roelfsema P, Changeux J-P, Dehaene S. Conscious Processing and the Global Neuronal Workspace Hypothesis. Neuron. 2020;105:776–98. doi: 10.1016/j.neuron.2020.01.026 32135090 PMC8770991

[69] Dehaene S, Sergent C, Changeux J-P. A neuronal network model linking subjective reports and objective physiological data during conscious perception. Proc Natl Acad Sci U S A. 2003;100:8520–5. doi: 10.1073/pnas.1332574100 12829797 PMC166261

[70] Brown R, Lau H, LeDoux JE. Understanding the Higher-Order Approach to Consciousness. Trends Cogn Sci. 2019;23:754–68. doi: 10.1016/j.tics.2019.06.009 31375408

[71] Lau H, Rosenthal D. Empirical support for higher-order theories of conscious awareness. Trends Cogn Sci. 2011;15:365–73. doi: 10.1016/j.tics.2011.05.009 21737339

[72] Dehaene S, Lau H, Kouider S. What is consciousness, and could machines have it? Science. 2017;358:486–92. doi: 10.1126/science.aan8871 29074769

[73] VanRullen R, Kanai R. Deep learning and the Global Workspace Theory. Trends Neurosci. 2021;44:692–704. doi: 10.1016/j.tins.2021.04.005 34001376

[74] Zeki S, Bartels A. Toward a Theory of Visual Consciousness. Conscious Cogn. 1999;8:225–59. doi: 10.1006/ccog.1999.0390 10448004

[75] Zeki S. The disunity of consciousness. Trends Cogn Sci. 2003;7:214–8. doi: 10.1016/s1364-6613(03)00081-0 12757823

[76] Zeki S. ■ REVIEW: Parallel Processing, Asynchronous Perception, and a Distributed System of Consciousness in Vision. Neuroscientist. 1998;4:365–72. doi: 10.1177/107385849800400518

[77] Cicurel R, Nicolelis MAL. The relativistic brain: how it works and why it cannot by simulated by a Turing machine. 2015.

[78] Köhler W. Gestalt psychology: an introduction to new concepts in modern psychology. New York: Liveright; 1992.

[79] Libet B. A Teastable Field Theory of Mind-Brain Interaction. J Conscious Stud. 1994;1:119–26.

[80] Libet B. Mind time: the temporal factor in consciousness. 1. Harvard Univ. Press paperback ed. Cambridge, Mass.: Harvard Univ. Press; 2005.

[81] McFadden J. The Conscious Electromagnetic Information (Cemi) Field Theory: The Hard Problem Made Easy? J Conscious Stud. 2002;9:45–60.

[82] McFadden J. Integrating information in the brain’s EM field: the cemi field theory of consciousness. Neurosci Conscious. 2020;2020. doi: 10.1093/nc/niaa016 32995043 PMC7507405

[83] Pockett S. NATURE OF CONSCIOUSNESS: a hypothesis. S.l.: IUNIVERSE COM; 2000.

[84] Oizumi M, Albantakis L, Tononi G. From the Phenomenology to the Mechanisms of Consciousness: Integrated Information Theory 3.0. Sporns O, editor. PLoS Comput Biol. 2014;10:e1003588. doi: 10.1371/journal.pcbi.1003588 24811198 PMC4014402

[85] Tononi G. Consciousness as Integrated Information: a Provisional Manifesto. Biol Bull. 2008;215:216–42. doi: 10.2307/25470707 19098144

[86] Tononi G, Boly M, Massimini M, Koch C. Integrated information theory: from consciousness to its physical substrate. Nat Rev Neurosci. 2016;17:450–61. doi: 10.1038/nrn.2016.44 27225071

[87] Searle JR. The Rediscovery of the Mind. First Edition edition. Cambridge, Mass: A Bradford Book; 1992.

[88] Searle JR. Minds, brains, and programs. Behav Brain Sci. 1980;3:417–24. doi: 10.1017/S0140525X00005756

[89] Searle JR. Is the Brain’s Mind a Computer Program? Sci Am. 1990;262:25–31. doi: 10.1038/scientificamerican0190-26 2294583

[90] Peirs C, Seal RP. Neural circuits for pain: Recent advances and current views. Science. 2016;354:578–84. doi: 10.1126/science.aaf8933 27811268 PMC11327866

[91] Bosking WH, Beauchamp MS, Yoshor D. Electrical Stimulation of Visual Cortex: Relevance for the Development of Visual Cortical Prosthetics. Annu Rev Vis Sci. 2017;3:141–66. doi: 10.1146/annurev-vision-111815-114525 28753382 PMC6916716

[92] Chen X, Wang F, Fernandez E, Roelfsema PR. Shape perception via a high-channel-count neuroprosthesis in monkey visual cortex. Science. 2020;370:1191–6. doi: 10.1126/science.abd7435 33273097

[93] Murphey DK, Yoshor D, Beauchamp MS. Perception Matches Selectivity in the Human Anterior Color Center. Curr Biol. 2008;18:216–20. doi: 10.1016/j.cub.2008.01.013 18258428

[94] Ham D, Park H, Hwang S, Kim K. Neuromorphic electronics based on copying and pasting the brain. Nat Electron. 2021;4:635–44. doi: 10.1038/s41928-021-00646-1

[95] Markram H, Muller E, Ramaswamy S, Reimann MW, Abdellah M, Sanchez CA, et al. Reconstruction and Simulation of Neocortical Microcircuitry. Cell. 2015;163:456–92. doi: 10.1016/j.cell.2015.09.029 26451489

